# Effect of Eu-implantation and annealing on the GaN quantum dots excitonic recombination

**DOI:** 10.1186/1556-276X-6-378

**Published:** 2011-05-09

**Authors:** Marco Peres, Sérgio Magalhães, Vincent Fellmann, Bruno Daudin, Armando José Neves, Eduardo Alves, Katharina Lorenz, Teresa Monteiro

**Affiliations:** 1 Departamento de Física e I3N, Universidade de Aveiro, Campus de Santiago, Aveiro, 3810-193, Portugal; 2 Instituto Tecnológico e Nuclear, Estrada Nacional 10, Sacavém, 2685-953, Portugal; 3 CEA/CNRS Group, "Nanophysique et Semiconducteurs", INAC, CEA/Grenoble, 17 rue des Martyrs, Grenoble Cedex 9, 38054, France; 4 CFNUL, Av. Prof. Gama Pinto, Lisboa, 1649-003, Portugal

## Abstract

Undoped self-assembled GaN quantum dots (QD) stacked in superlattices (SL) with AlN spacer layers were submitted to thermal annealing treatments. Changes in the balance between the quantum confinement, strain state of the stacked heterostructures and quantum confined Stark effect lead to the observation of GaN QD excitonic recombination above and below the bulk GaN bandgap. In Eu-implanted SL structures, the GaN QD recombination was found to be dependent on the implantation fluence. For samples implanted with high fluence, a broad emission band at 2.7 eV was tentatively assigned to the emission of large blurred GaN QD present in the damage region of the implanted SL. This emission band is absent in the SL structures implanted with lower fluence and hence lower defect level. In both cases, high energy emission bands at approx. 3.9 eV suggest the presence of smaller dots for which the photoluminescence intensity was seen to be constant with increasing temperatures. Despite the fact that different deexcitation processes occur in undoped and Eu-implanted SL structures, the excitation population mechanisms were seen to be sample-independent. Two main absorption bands with maxima at approx. 4.1 and 4.7 to 4.9 eV are responsible for the population of the optically active centres in the SL samples.

## Introduction

Self-assembled GaN quantum dots (QD) stacked in superlattices (SL) with AlN spacer layers are known to be important nanostructures for optoelectronic applications in the UV/visible and infrared spectral regions [1-3]. The GaN QD excitonic recombination is usually characterized by a broad band recombination with ca. 300 meV of full width at half maximum for samples with homogeneous dot size distribution [3]. It is well established that the GaN QD excitonic recombination can occur at photon energies above and below the GaN bulk bandgap [1-8]. This behaviour is driven by the combined effects of the quantum confinement (QC) of the carriers and the quantum confined Stark effect (QCSE), which is influenced by the strain state of the stacked heterostructures [4,8,9]. The peak position of the GaN QD excitonic recombination is also known to be very sensitive to the dot size, shape and thermal annealing treatments [3-10]. In addition, and despite the expected thermal stability of the QD photoluminescence (PL) intensity, non-radiative processes described by different activation energies have been reported in undoped and intentionally doped SL structures [3,6,11-14]. Indeed, the low temperature to room temperature PL intensity ratio, *I*(14 K)/*I*(RT), exhibits a sample dependent behaviour [3,6,11-14], which needs further investigation. Therefore, it is an aim of this article to address the issue of the excitation and de-excitation mechanisms of the emission of as-grown, thermally annealed and Eu-implanted GaN QD embedded in AlN spacers.

As-grown, annealed and Europium implanted and annealed GaN QD/AlN SL were studied by temperature-dependent PL and photoluminescence excitation (PLE) in order to analyse the influence of the excitation population mechanisms on the PL efficiency of the excitonic GaN QD recombination. The excitation paths were seen to be sample independent while different PL emission bands were detected for the non-doped and Europium doped SL. The effects of the implantation fluence as well as its relationship with the carrier localization in the GaN QD will be discussed.

## Experimental

The GaN QD/AlN SL structures were grown by molecular beam epitaxy (MBE) on AlN/Al_2_O_3 _pseudo substrates as described elsewhere [15]. The investigation was performed on three sets of samples consisting of 10 (#1110) and 20 (#987 and #989) nm stacks of (0001) GaN QD with AlN interlayers of 30 (#1110) and 13 (#987 and #989) nm. The QD height has been set around 3.0 (#1110), 3.7 (#987) and 4.2 (#989) nm from growth deposition parameters, in accordance with previous reported optical experiments [4] and theoretical predictions [8,9]. An AlN cap layer was furthermore grown on the SL top part (270 nm for sample #1110 and 30 nm for samples #987 and #989). The GaN QD density and diameter was estimated to be in the 10^11 ^cm^-2 ^and 15 to 20 nm ranges, respectively [16]. The as-grown sample #1110 was further submitted to thermal annealing treatments at 1200°C in flowing N_2 _at 1 mbar pressure and placing a piece of AlN/sapphire face-to-face with the samples as a proximity cap to protect the surface during the high temperature treatment. The #987 and #989 GaN QD/AlN SL were implanted with high (1 × 10^14-15 ^ions cm^-2^) and low (1 × 10^13 ^ions cm^-2^) fluences of Europium ions; the SL structures were further submitted to post-implantation thermal annealing in order to achieve Eu^3+ ^optical activation [17,18].

Steady-state PL measurements were carried out between 14 K and room temperature (RT) using for excitation photons with energy of 3.81 and 4.7 eV corresponding to the 325 nm line of a cw He-Cd laser (excitation density less than 0.6 W cm^-2^) and a monochromated 1000 W Xe lamp, respectively. The spot size of the two light sources was 1 and 5 mm in diameter, so in both cases the luminescence arises from a large number of QDs. The used excitation energies are below the AlN bandgap (approx. 6 eV). The samples were mounted in the cold finger of a closed-cycle helium cryostat and the sample temperature was controlled in the range from 14 K up to RT. The luminescence was measured using a Spex 1704 monochromator (1 m, 1200 mm^-1^) fitted with a cooled Hamamatsu R928 photomultiplier tube. For the PLE measurements, the emission monochromator was set at the GaN QD excitonic recombination and the excitation wavelength was scanned up to 5.2 eV. The spectra were corrected to the lamp and optics.

X-ray reflection (XRR) was performed on a high-resolution system using a Göbel mirror to focus the beam and CuKα_1,2 _radiation.

## Results and discussion

**Figure 1:**
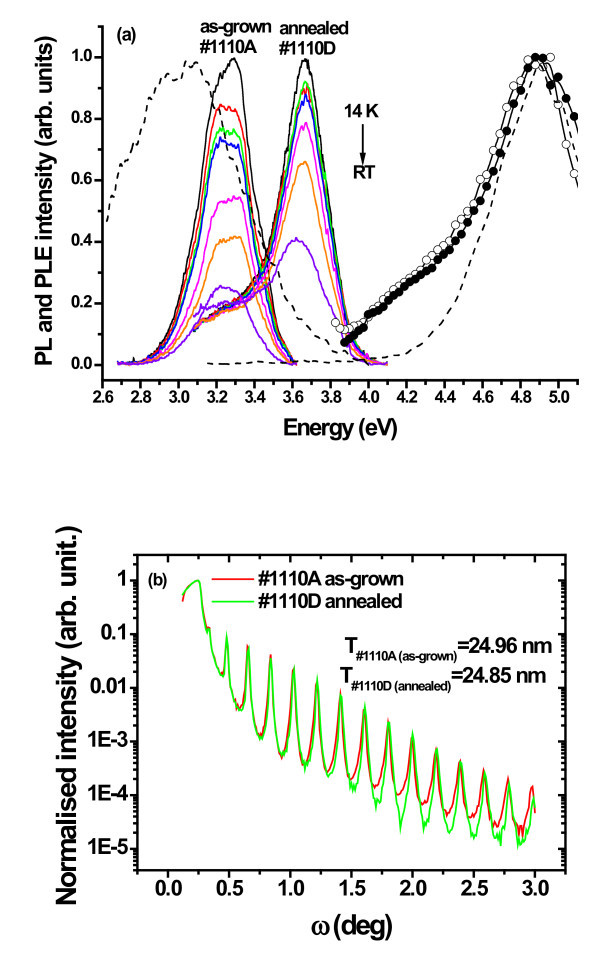
**Influence of the thermal annealing on the optical (PL and PLE) and XRR properties of GaN QD/AlN SL structures**. **(a) **Temperature-dependent PL spectra of the excitonic recombination for a 10-period GaN QD/AlN SL structure before and after thermal annealing at 1200°C (full lines). Normalised RT PLE spectra monitored at the PL band maximum for the as-grown (line + closed symbols) and annealed (line + open symbols) samples. 14 K PL and PLE spectra of an AlN layer (dashed lines). **(b) **Specular X-ray reflection for the as-grown and annealed GaN QD/AlN SL structures. The XRR determined period thickness is shown in the graph for both samples.

Figure 1 shows the temperature-dependent PL spectra of a 10-period as-grown and thermally annealed GaN QD/AlN SL (#1110A and #1110D, respectively). The main maxima of the GaN QD excitonic recombination occur below (before thermal annealing) and above (after thermal annealing) the bulk GaN bandgap (approx. 3.5 eV). This suggests that the annealing of the SL structure at 1200°C in nitrogen promotes a change in the balance between the QC and QCSE as seen by the high-energy shift of the GaN QD recombination [7]. Among the various effects which could be responsible for the blue shift of the PL peak position, both interdiffusion and thermally-induced strain relaxation mechanism should be considered to explain the competition between the QC and QCSE [7]. The large number of satellites observed by XRR (Figure 1b) indicates that both the as-grown and the annealed SL have smooth interfaces and high-crystalline quality. The PL thermal quenching measured between 14 K and RT is mediated by different non-radiative processes as indicated by an intensity ratio, *I*(14 K)/*I*(RT), of 4 and 2.5, respectively, for the as-grown and annealed samples, when excited with the He-Cd laser line. This ratio is usually a measure of the carrier localization on the QD and it can be concluded that a higher PL thermal stability was achieved after the thermal annealing.

On the right side of Figure 1a, the RT PLE spectra for both samples show the same excitation paths for the GaN QD excitonic recombination. This means that independent of the annealing effects the population mechanisms, which give rise to the GaN QD emission, are identical. A large asymmetric broad absorption band with a shoulder at approx. 4.1 eV extends to higher energies showing a maximum between 4.7 and 5.0 eV. The low and high energy absorption bands were also observed by others authors [12,19] and have been assigned to the excited state absorption from the GaN QD and to the absorption by the wetting layer [12,19,20]. As our SL systems have AlN spacer layers, we must also account for potential excitation mechanisms via the AlN host. In particular, it is well established that oxygen-related defects in AlN samples are optically active and give rise to absorption and emission bands in the ultraviolet spectral region [21]. In Figure 1a, the PL and PLE spectra of an undoped AlN layer is shown for comparison. The oxygen-related emission [21] with a maximum at approx. 3.0 eV is observed under excitation with photons of approx. 4.9 eV energy. Despite the fact that the AlN layer PL band partially overlaps with the one of the SL structures, their spectral shapes and peak position are clearly distinct, which means that they are obviously due to different transitions. On the contrary, the high-energy absorption band detected on the PLE spectra monitored on the band maxima of the GaN QD excitonic recombination overlaps with the one associated to the oxygen defect on the AlN layer, suggesting that the GaN QD emission band could also be fed by the defect level from AlN spacers, buffer or capping layer in the SL structures.

**Figure 2:**
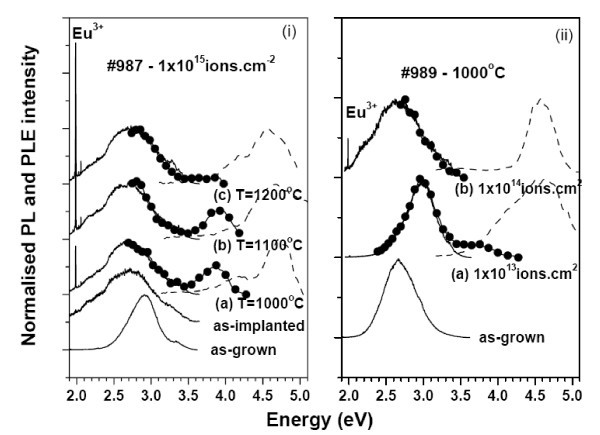
**Normalised 14 K PL and PLE spectra for the as-grown and Eu-implanted GaN QD/AlN SL structures**. Black full lines: PL spectra obtained with excitation with photons of 3.81 eV energy (He-Cd laser line); lines and closed symbols: PL spectra obtained with excitation of 4.7 eV. Dashed lines: PLE spectra monitored at the maxima of the low energy broad emission band. (i) stands for sample 987 which was implanted with 1 × 10^15 ^atoms.cm^-2 ^and annealed at 1000°C (a), 1100°C (b) and 1200°C (c). (ii) stands for sample 989 which was implanted with 1 × 10^13 ^atoms.cm^-2 ^(a) and 1 × 10^14 ^atoms.cm^-2 ^(b) and annealed at 1000°C.

Two other sets of 20 periods GaN QD/AlN SL (#987 and #989) with larger quantum dots (average QD heights of 3.7 and 4.2 nm according to [9]) were implanted with different fluences of Eu^3+ ^ions. The SL structures were further submitted to thermal annealing treatments between 1000 and 1200°C in order to achieve Europium optical activation. The Eu^3+ ^emission from GaN QD as well as from AlN layers were identified previously [17,18]. The structural analysis by X-ray diffraction (XRD) of the implanted and annealed SL structures showed that high implantation fluences (10^14 ^and 10^15 ^ions cm^-2^) lead to higher lattice damage causing an expansion of the SL structure in the [0001] direction, while lower fluence does not change the XRD characteristics of the sample [17]. For these samples, and besides the Eu^3+ ^luminescence, additional broad emission bands can be identified on the high energy side, as shown in Figure 2i, ii. Independently of the annealing temperature, a very broad emission band peaked at approx. 2.7 eV could be observed under excitation with photons of 3.81 eV energy for samples implanted with high fluence. The similarity of the spectral shape and peak position of the broad band with the emission detected under the same excitation conditions in the as-implanted sample indicates that it arises from large, 'blurred' GaN QD present in the damaged region of the implanted SL. The PLE spectra monitored at 2.7 eV is similar to the one shown in Figure 1a for the non-implanted SL samples suggesting that the optically active defects in the implanted SL are excited via the same paths. This is also confirmed with wavelength-dependent PL studies as seen in Figure 2. Exciting the samples in the wetting layer and/or oxygen-related AlN defect absorption bands (approx. 4.7 eV) makes the 2.7-eV PL always observable. Besides the 2.7-eV emission band, the GaN QD/AlN SL structures implanted with high fluence show an additional emission band peaked at 3.9 eV under 4.7 eV excitation. The observation of two GaN QD emission bands suggests the presence of a bimodal size distribution in the studied SL. Bimodality of GaN QD population in similar SL structures was previously reported by Adelmann et al. [16] and they found that such distribution occur at high GaN coverage and/or substrate temperature, which is the case of the analysed SL samples.

**Figure 3:**
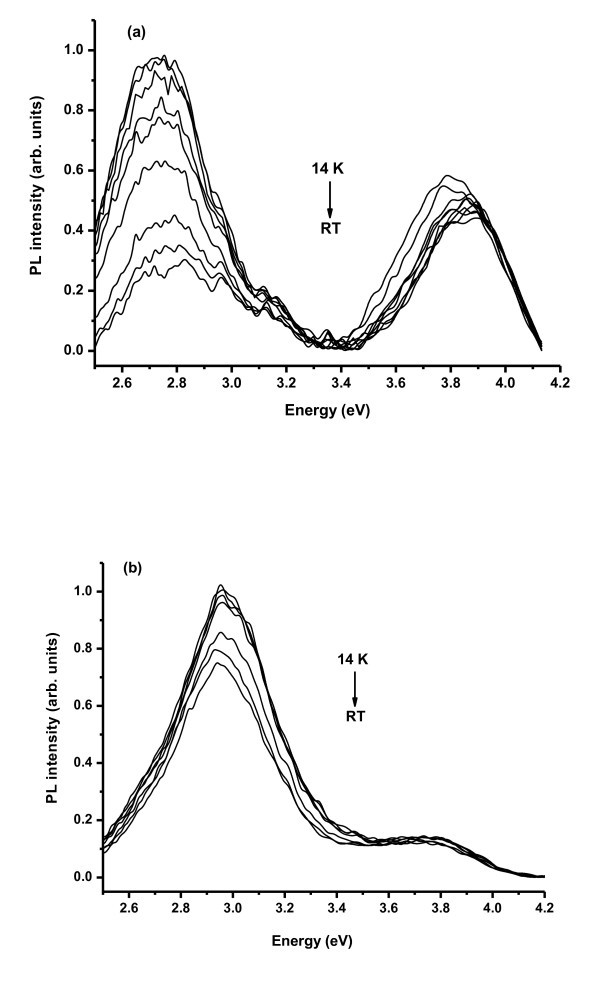
**Temperature-dependent PL spectra: **for sample **(a) **#987 implanted with 1 × 10^15 ^atoms.cm^-2 ^and annealed at 1100°C and **(b) **#989 implanted with 1 × 10^13 ^atoms.cm^-2 ^and annealed at 1000°C obtained with 4.7 eV excitation.

Figure 3a shows typical temperature-dependent PL spectra of both optical emitting centres (2.7 and 3.9 eV bands) observed with excitation with photons of 4.7 eV energy. A fast decrease of the intensity of the 2.7-eV emission is seen with increasing temperature from 14 K to RT (*I*(14 K)/*I*(RT)~4). On the contrary, the high energy emission peak at 3.9 eV is seen to have an intensity which is nearly constant up to 200 K. A slight increase of the PL intensity was seen for higher temperatures accompanied with a small energy shift of the peak position, commonly observed in small GaN QDs [11]. For the SL implanted with high fluence only, the GaN QD PL band due to the larger blurred GaN QD have strong non-radiative de-excitation processes likely to be due to the defects generated by ion implantation.

For samples implanted with lower fluence (#989(a)--Figures 2ii and 3b), a narrower GaN QD exciton recombination could be detected, for the 3.0-eV PL when the SL is excited either with 3.81 and 4.7 eV photons energy. Compared with the GaN QD PL detected in the as-grown SL, the emission band is shifted to higher energy similar to the case of undoped annealed high-structural quality SL shown in Figure 1. The absence of the large broad emission at 2.7 eV is consistent with the high-structural quality of this sample where no lattice expansion was found as confirmed by XRD [17]. For this SL sample also, a bimodal GaN QD distribution is present as shown by the observation of two emitting bands from GaN QD at approx. 3.0 and 3.8 eV. Figure 3b shows the temperature-dependent PL of both optical centres observed with 4.7 eV excitation. As observed for the high fluence implanted SL, the intensity ratio *I*(14 K)/*I*(RT) of the high energy GaN QD emission is practically constant up to RT while for the 3.0 eV PL band a ratio of 1.3 was found. The small thermal quenching of the luminescence observed for the SL implanted with lower fluence suggests that the competing non-radiative processes are less important as expected for the lower damaged SL structure.

## Conclusions

In Eu-implanted SL structures, the GaN QD recombination was found to be dependent on the implantation fluence. For samples implanted with high fluence, a broad emission band at 2.7 eV was tentatively assigned to the emission occurring at large blurred GaN QD. The temperature-dependent PL analysis in this sample evidences a fast decrease of the luminescence, consistent with the competing non-radiative relaxation processes expected for a large defective SL. This emission band is absent in the lower fluence implanted SL structure, which has high structural quality. In this case, the GaN QD PL at approx. 3.0 eV evidences a smaller thermal quenching with increasing temperatures from 14 K to RT. Additionally, the peak position of the emission shifts to higher energy when compared with the one of the as-grown sample. This blue shift was also observed in undoped and annealed SL showing that a change in the balance between the QC and QCSE occur with thermal annealing treatments.

Despite the fact that different de-excitation processes occur in as-grown, annealed and Eu-implanted SL, the optically active centres in the GaN QD/AlN SL are excited via the same paths: two main absorption bands with maxima at approx. 4.1 and 4.7 to 4.9 eV.

## Abbreviations

MBE: molecular beam epitaxy; PL: photoluminescence; PLE: photoluminescence excitation; QCSE: quantum confined Stark effect; QD: quantum dots; RT: room temperature; SL: superlattice; XRR: X-ray reflection.

## Competing interests

The authors declare that they have no competing interests.

## Authors' contributions

All the authors have made substantial intellectual contributions to the presented study. VF and BD were responsible for the growth of the analysed samples, SM, EA and KL performed the implantation and annealing treatments and carried the experiments and data analysis of the structural samples characterization, MP, AJN and TM carried out the acquisition of the optical data and their interpretation. All the authors have together discussed and interpreted the results. All the authors read and approved the final manuscript.
